# Ligand Installation to Polymeric Micelles for Pediatric Brain Tumor Targeting

**DOI:** 10.3390/polym15071808

**Published:** 2023-04-06

**Authors:** Takayoshi Watanabe, Hayato Laurence Mizuno, Jumpei Norimatsu, Takumi Obara, Horacio Cabral, Kouhei Tsumoto, Makoto Nakakido, Daisuke Kawauchi, Yasutaka Anraku

**Affiliations:** 1 Department of Bioengineering, Graduate School of Engineering, The University of Tokyo, 7-3-1 Hongo, Bunkyo-ku, Tokyo 113-8656, Japan; 2 Department of Biochemistry and Cellular Biology, National Center of Neurology and Psychiatry (NCNP), Tokyo 187-8551, Japan; 3 Department of Chemistry and Biotechnology, School of Engineering, The University of Tokyo, Tokyo 113-8654, Japan; 4 Medical Proteomics Laboratory, The Institute of Medical Science, The University of Tokyo, Tokyo 113-8654, Japan; 5 Department of Materials Science and Engineering, School of Materials and Chemical Technology, Tokyo Institute of Technology, Tokyo 152-8550, Japan

**Keywords:** CD276, medulloblastoma, micelles, ligand installation, anti-CD276 antibody

## Abstract

Medulloblastoma is a life-threatening disease with poor therapeutic outcomes. In chemotherapy, low drug accumulation has been a cause of these outcomes. Such inadequate response to treatments has been associated with low drug accumulation, particularly with a limited cellular uptake of drugs. Recently, the conjugation of drugs to ligand molecules with high affinity to tumor cells has attracted much attention for enhancing drug internalization into target cells. Moreover, combining tumor-targeting ligands with nano-scaled drug carriers can potentially improve drug loading capacity and the versatility of the delivery. Herein, we focused on the possibility of targeting CD276/B7-H3, which is highly expressed on the medulloblastoma cell membrane, as a strategy for enhancing the cellular uptake of ligand-installed nanocarriers. Thus, anti-CD276 antibodies were conjugated on the surface of model nanocarriers based on polyion complex micelles (PIC/m) via click chemistry. The results showed that the anti-CD276 antibody-installed PIC/m improved intracellular delivery into CD276-expressing medulloblastoma cells in a CD276-dependent manner. Moreover, increasing the number of antibodies on the surface of micelles improved the cellular uptake efficiency. These observations indicate the potential of anti-CD276 antibody-installed nanocarriers for promoting drug delivery in medulloblastoma.

## 1. Introduction

Medulloblastoma (MB) is the most typical malignant brain tumor in the pediatric population. It comprises 20% of all childhood brain tumors and 63% of intracranial embryonal tumors [1]. Known for its high lethality and 5-year survival rate of 40–60% [2], an effective yet safe treatment strategy is urgent [3]. State-of-the-art treatment is mostly based on surgical intervention [4], with a combination of adjuvant radiotherapy [5] and/or chemotherapy [6]. Though such treatments have shown a certain level of success, the treatment effect is unsatisfactory in a large population [7], who frequently suffer symptoms generally termed “chemobrain,” caused by the side effects of the administered antitumor drugs [8]. These side effects are thought to be a product of drugs being unintendedly delivered to healthy brain tissue due to their poor tumor-targeting effect [9]. One such drug is Topotecan (TPT), which is known to accumulate poorly at a rate of approximately 0.5% dose/g tumor [10]. Given the example of TPT, it is clear that a strategy to target and deliver drugs to the tumor at high efficiency is in high demand to improve the clinical outcomes of MB while minimally risking the patient’s quality of life.

To improve the therapeutic outcomes of chemotherapy, various approaches to targeting tumor cells and delivering sufficient drug payload have been explored so far [11]. Recently, conjugating antibodies or fragmented antibodies that bind specifically to proteins highly expressed in tumor cells to drugs has provoked attention in the field of drug delivery [12]. One strategy example is conjugating an antibody with high affinity to CD276, a transmembrane protein highly expressed in pediatric tumors [13], to an antitumor drug Cu-NOTA [14]. Here, drug accumulation within breast cancer cells was significantly improved because of the endocytosis of an anti-CD276 antibody (CD276Ab) by tumor cells, followed by its subcellular localization in both early endosomes and lysosomes [15], thereby demonstrating the effectivity of utilizing antibodies to drive the drugs to the tumor. In another set of studies, nanocarriers have been used to deliver the carried drugs across the blood–brain tumor barrier (BBTB) [16,17,18]. An illustrative instance of the implementation of this approach was demonstrated using cRGD conjugated polymeric micelle for glioblastoma targeting [19]. As such, utilizing the tumor-targeting effect of antibodies and nanocarriers could offer efficient delivery of drugs to the intended cells, benefiting both the effectiveness and safety of chemotherapy.

**Figure 1:**
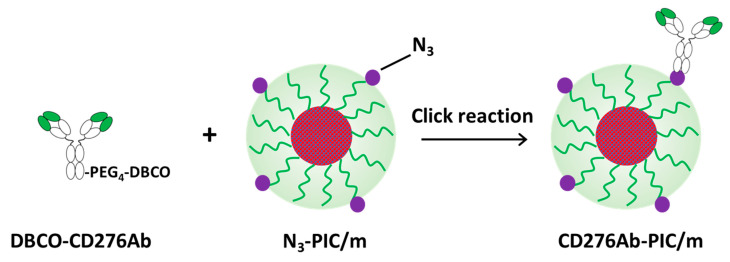
CD276Ab installation to N_3_-PIC/m by click reaction.

Polyion complex micelle (PIC/m) composed of oppositely charged polymers is a nanocarrier that can sufficiently withstand the ionic strength of solvents [20,21] and environmental pH [22]. Therefore, it works as a model carrier because of its high stability. In this pilot study, influenced by the past success of antibody-drug conjugates and tumor-targeted nanocarriers, fluorescently-labeled model micelles were modified with multiple anti-CD276 antibodies (CD276Ab), then tested for their efficiency in targeting tumor-derived cells (Figure 1). As a proof of concept, CD276Ab was introduced to fluorescently-labeled azide (N_3_)-equipped polyion complex micelles (N_3_-PIC/m) through click chemistry, then added to MB patient-derived DAOY cell cultures [23]. The study results indicated that the antibodies were successfully introduced to the surface of the PIC/m, which elicited a significant improvement in the rate of internalization of the micelle by MB cells *in vitro*. These findings suggest that the proposed strategy can potentially improve drug delivery efficiency to tumor cells, advancing the design of effective and safe treatment for MB.

## 2. Materials and Methods

### 2.1. Materials

Dibenzocyclooctyne-poly(ethylene glycol)_4_-*N*-hydroxysuccinimidyl ester (DBCO-PEG_4_-NHS) was purchased from Click Chemistry Tools Co. Inc. (Scottsdale, AZ, USA). The 1-ethyl-3-(3-dimethylaminopropyl) carbodiimide hydrochloride (EDC/HCl) and dimethyl sulfoxide (DMSO) were purchased from Tokyo Chemical Industry Co., Ltd. (Tokyo, Japan). Fluorescamine, sodium dihydrogenphosphate dihydrate, disodium hydrogenphosphate, hydrochloric acid, 4% paraformaldehyde phosphate buffer solution, and Dulbecco’s phosphate buffered saline (D-PBS(-)) were purchased from FUJIFILM Wako Pure Chemical Co. (Tokyo, Japan). Ab SpinTrap Protein G Sepharose High Performance was purchased from Cytiva Co. (Marlborough, MA, USA). Coverglass chamber 8 well was purchased from AGC Techno Glass (Shizuoka, Japan). Sulfo-Cyanine 5 NHS ester was purchased from Lumiprobe (Hunt Valley, MD, USA). Pierce Bovine Serum Albumin Standard Ampules, DyLight 488 NHS Ester, Dulbecco’s modified Eagle Medium (DMEM) with High Glucose and Penicillin-Streptomycin (10,000 U/mL) were purchased from Thermo Fisher Scientific Co. (Waltham, MA, USA). Cellstain Hoechst 33258 solution was purchased from Dojindo Kagaku Co. (Kumamoto, Japan). Tris(hydroxymethyl) aminomethane and Glycine Aminoacetic Acid were purchased from Nacalai Tesque Inc. (Kyoto, Japan). Fetal Bovine Serum was purchased from Sigma-Aldrich Co. (St. Louis, MO, USA). The DAOY (HTB-186) cell line was purchased from ATCC (Manassas, VA, USA).

### 2.2. IgG Preparation as Recombinant Proteins

The amino acid sequence of the 8H9 antibody was obtained from the PDB database (PDB ID: 5CMA). The IgG antibody was expressed and purified as described previously [24]. Briefly, Expi CHO cells were co-transfected with expression vectors for heavy and light chains, and the supernatant was collected 12 days after transfection. The supernatant was purified via affinity chromatography using rProtein A Sepharose resin and subsequent size exclusion chromatography. The purity of the eluted sample was evaluated by SDS-PAGE followed by Coomassie staining.

### 2.3. Estimation of Number of DBCO Conjugated to CD276Ab by Fluorescamine

The number of residual primary amine groups per antibody was determined through a previously reported assay method using fluorescamine [25,26]. First, fluorescamine was dissolved in DMSO at 3.0 mg/mL and mixed thoroughly. A 2.0 mg/mL bovine serum albumin (BSA) standard sample in 0.9% saline and 0.05% sodium azide solution was adjusted to 7.8, 15.6, 31.2, 62.5, 125, 250, and 500 μg/mL through multistep dilution with D-PBS(-). Then 9 μL of each BSA solution was mixed with 3 μL fluorescamine solution and reacted for 15 min at 25 °C, and 2 μL of each solution was used to measure the fluorescence intensity to draw a standard curve. Note here it has been reported that BSA has 30 residual primary amine groups per molecule [27]. For the estimation of the primary amine groups in the CD276Ab solution, 3 μL of the CD276Ab solution (0.10 mg/mL) in D-PBS(-) was mixed with fluorescamine solution (3.0 mg/mL) in DMSO and reacted under the abovementioned conditions.

Estimation of the number of DBCO groups conjugated onto CD276Ab antibodies was conducted by measuring the remaining residual amine groups after DBCO conjugation, then comparing this to that of ‘*as prepared*’ CD276Ab antibodies. 0, 2.8, 10.5, 22.1, 88.6, and 177.1 μg/mL NHS-PEG_4_-DBCO solution (2 μL) in DMSO was mixed with CD276Ab solution (9 μL, 0.10 mg/mL) in D-PBS(-) and left still at 25 °C for 1 h. Here, the amount of each prepared NHS-PEG_4_-DBCO corresponds to 0, 0.03, 0.13, 0.25, 1.0, and 2.0, equivalent to the number of residual primary amines in the CD276Ab solution, respectively. Fluorescamine solution (3 μL, 3.0 mg/mL) was added and reacted for 15 min at 25 °C (N = 5). The number of residual primary amine and conjugated DBCO were estimated from the standard curve drawn using a BSA solution.

### 2.4. Fabrication of DBCO-CD276Ab and DBCO-CD276Ab-Cy5

DBCO-PEG_4_-NHS was first conjugated to the amine groups of CD276Ab. DBCO-PEG_4_-NHS in DMSO (3.3 μL, 10.0 mg/mL) was added to the antibody solution (600 μL, 1.12 mg/mL) in D-PBS(-) at concentrations equivalent to 0.25 times the number of amine groups within the solution and incubated at room temperature for 1 h.

Fabrication of DBCO-CD276Ab-Cy5 was conducted using the DBCO-CD276Ab prepared above. Cy5-NHS ester solution (3.3 μL, 5.0 mg/mL) in DMSO was added to DBCO-CD276Ab solution (603.3 μL) in D-PBS(-), then incubated at room temperature for 15 min at 25 °C.

### 2.5. Purification of DBCO-CD276Ab and DBCO-CD276Ab-Cy5

DBCO-CD276Ab fabricated through the aforementioned scheme was purified via a Protein G HP SpinTrap (Cytiva, Marlborough, MA, USA) [28]. The column was initially washed with phosphate buffer (20 mM, pH 7.0, 0 mM NaCl) 3 times without a lid, then the crude mixture was loaded to the column and left still for 4 min. Subsequently, the column was washed again with the same buffer, then 400 μL of 0.1 M glycine-HCl (pH 2.7) was loaded for elution. Tris-HCl (17 μL, 1 M, pH 9.0) was added to the microcentrifuge tube, which holds the column for neutralization so that the pH of the DBCO-CD276Ab solution after neutralization was 7.0. After centrifugation (2000 rpm, 20 s), DBCO-CD276Ab was collected from the microcentrifuge tube at a yield of 50.0% (N = 3). The concentration of purified antibodies was calculated by measuring the absorbance at 280 nm (Figure S1, see Supplementary Materials). The same protocol was used to purify DyLight 488-(DBCO-CD276Ab-DyLight 488) or Cy5-labeled (DBCO-CD276Ab-Cy5) antibodies.

### 2.6. Immunocytochemical Evaluation of DBCO-CD276Ab Functionality

#### 2.6.1. DAOY Cell Culture

DAOY (HTB-186) cell line was cultivated with high glucose-supplemented DMEM plus 10% FBS, 100 U/mL penicillin, and 100 µg/mL of streptomycin. The cells were maintained under optimal conditions (e.g., 5% CO_2_ at 37 °C in a humidified atmosphere) and subcultured when the confluency reached 70–80%. pCMV Greenfire lentiviral plasmid encoding *EGFP* and firefly *Luciferase* were used for GFP labeling of DAOY cells in some experiments.

#### 2.6.2. CD276 Detection by Flow Cytometry

DAOY cells were harvested at a confluency of 80% and were resuspended with PBS supplemented with 10% FBS (FACS Buffer). A total of 500 cells were stained in 100 µL of FACS buffer containing the CD276 antibody conjugated with phycoerythrin (PE) (Biolegend, #331605; 1:200 dilution) for 1 h at 4 °C (Figure S2, see Supplementary Materials). FACS was performed using BD FACSCanto II (BD Biosciences) immediately after washing the cells with FACS Buffer. Data were analyzed using FlowJoTMv10 (BD Biosciences).

#### 2.6.3. Immunocytochemical Evaluation

The concentration of purified Cy5-labeled DBCO-CD276Ab was determined by measuring the absorption at 280 nm by NanoDrop One (Thermo Scientific, Waltham, MA, USA) and diluted to 5.0 μg/mL with D-PBS(-). GFP-expressing DAOY cells (1.5 × 10^4^ cells/well) were seeded on an 8-well chamber and then incubated in DMEM supplemented with 10% FBS, 100 U/mL penicillin, and 100 µg/mL of streptomycin at 37 °C, 5% CO_2_, until 70% confluency. CD276Ab (200 μL) was added to the cells at 0, 12.5, 25.0, 50.0, and 100 μg/mL, followed by subsequent incubation for 1 h at 37 °C, 5% CO_2_. Each concentration was adjusted by multistep dilution using a culture medium. After washing the cells three times with D-PBS(-), DBCO-CD276Ab-Cy5 (200 μL, 30.0 μg/mL) was added to the cell cultures and incubated for 1 h at 37 °C and 5% CO_2_. The cell nuclei were counterstained with Hoechst 33258, then the cells were observed, and the images were captured using a confocal laser scanning microscope (LSM780-06, Carl Zeiss, Oberkochen, Germany). The total fluorescence intensity of Cy5 within the cell cytoplasm (determined based on GFP fluorescence) of each cell was calculated and compared amongst the different culture conditions (*n* = 50). All image analyses were conducted using Image J [29,30]. A significant difference in fluorescence intensity between culture conditions was detected by performing a Student’s *t*-test followed by Bonferroni correction for multiple comparisons.

### 2.7. Fabrication of N_3_-PIC/m

N_3_-PIC/m was prepared based on a previous report [31]. Briefly, an aniomer solution was prepared by dissolving Methoxy-PEG-poly (α,β-aspartic acid) (PEG-PAsp, 2k-75), PEG-PAsp-Cy5 (2.2k-73), and N_3_-PEG-PAsp (2k-68) in phosphate buffer (10 mM, pH 7.4, 0 mM NaCl) at a ratio of 20:40:40, respectively, so that the final polymer concentration was 1.0 mg/mL. This mixing ratio was used to prepare N_3_ groups on 20% of the product N_3_-PIC/m’s surface area. A catiomer solution was prepared by dissolving Methoxy-PEG-poly[(5-aminopenthy)-α,β-aspartamide] (2k-72) in phosphate buffer (10 mM, pH 7.4, 0 mM NaCl) at 1 mg/mL. The polymer solutions were mixed at an aniomer:catiomer ratio of 5:4 to fabricate N_3_-PIC/m. The product micelles were stabilized by cross-linking the polymers with EDC/HCl, then purified with an ultracentrifugation unit, Vivaspin 6 MWCO 100,000 Da (Sartorius Stedium Biotech GmbH, Goettingen, Germany), at 2000 rpm. The final micelle solution was prepared in D-PBS(-), and its concentration was determined by measuring and comparing the Cy5-derived fluorescence with a standard curve. The characterization of N_3_-PIC/m was conducted via dynamic light scattering (DLS) measurement (Zetasizer Nano ZS90, Malvern Panalytical Ltd., Malvern, UK).

### 2.8. Fabrication of CD276Ab-PIC/m and DyLight 488-Labeled CD276Ab-PIC/m

DBCO-CD276 or DBCO-CD276-DyLight 488 (93.8 μL, 156.1 μg/mL) in a mixture of Gly-HCl and Tris-HCl was added to N_3_-PIC/m (100 μL, 0.24 mg/mL) in phosphate buffer (10 mM, pH 7.4, 0 mM NaCl) [32] at a ratio equivalent to 0–2.3 times the number of N_3_ moiety, then reacted overnight at 25 °C. CD276Ab-PIC/m was purified by gel permeation chromatography (GPC) (JASCO, Tokyo, Japan) equipped with a Superdex 200 increase 10/300 GL column (GE Healthcare, Chicago, IL, USA), and the yield was 40% (N = 3). Using this method, unreacted CD276Ab was removed (Figure S3, see Supplementary Materials). The diffusion coefficient and the number of CD276Ab installed to N_3_-PIC/m were calculated through fluorescence correlation spectroscopy (FCS). As for the FCS, the diffusion time τ of Cy5 and DyLight 488 were measured. This value can be expressed using confocal ratio ω and diffusion coefficient D as in Equation (1) [33]. The diffusion coefficient of CD276Ab-PIC/m, *D*_CD276Ab-PIC/m_, was calculated using the diffusion coefficient of Cy5, $(3.7\pm 0.15)\times 10^{-6}\;{cm}^{2}/s$ [34], and Equation (2) at the same confocal ratio. The diameter of PIC/m and CD276Ab-PIC/m were calculated using Equation (3) [33]. In the equation, diffusion coefficient D can be expressed using the Stokes-Einstein equation, where *k* is the Boltzmann constant, *T* is the temperature, *η* is the viscosity, and *r* is the hydrodynamic radius [29].

(1) $$\tau =\frac{\omega }{D}$$

(2) $$\frac{\tau_{Cy5-NHS}}{\tau_{CD276Ab-PIC/m}}=\frac{D_{CD276Ab-PIC/m}}{D_{Cy5-NHS}}$$

(3) $$D=\frac{kT}{6\pi \eta r}$$

Counts per molecule, $\eta^{\prime }$, reflects the fluorescence intensity of each fluorescent molecule. The number of CD276Ab installed to N_3_-PIC/m and the number of Cy5 molecules per CD276Ab-PIC/m were calculated using Equations (4) and (5) [35].

(4) $$\mathrm{Installation}\;\mathrm{number}=\frac{{\eta^{\prime }}_{CD276Ab-PIC/m}}{{\eta^{\prime }}_{DBCO-CD276Ab}}$$

(5) $$\mathrm{Cy}5\;\mathrm{bearing}\;\mathrm{number}=\frac{{\eta^{\prime }}_{CD276Ab-PIC/m}}{{\eta^{\prime }}_{Cy5-NHS}}$$

where *η*′_CD276Ab-PIC/m_, *η*′_DBCO-CD276Ab_, and *η*′_Cy5-NHS_ denotes the counts per molecule of CD276Ab-PIC/m, DBCO-CD276Ab, and Cy5-NHS, respectively.

### 2.9. Cytotoxicity of CD276Ab-PIC/m

GFP-expressing DAOY cells (1.0 × 10^4^) were seeded on each well of an 96-well plate and then cultured in DMEM supplemented with 10% FBS, 100 U/mL penicillin, and 100 µg/mL of streptomycin at 37 °C, 5% CO_2_ until the chamber bottom reached 70% confluency. 50 μL of N_3_-PIC/m, CD276Ab(1)-PIC/m, and CD276Ab(2)-PIC/m (10 µg/mL) was added, then incubated in the culture medium for 6 h at 37 °C, 5% CO_2_. After washing the cells three times with D-PBS, the GFP-derived fluorescence was quantified via a multiplate reader (Spark, Tecan, Männendorf, Switzerland) at 478 nm excitation and 535 nm detection. The fluorescence of cells incubated in the growth medium was considered 100% viable and used to standardize the viability of those incubated with the PIC/m.

### 2.10. Cell Internalization Efficiency of CD276Ab-PIC/m

GFP-expressing DAOY cells (1.5 × 10^4^) were seeded on each well of an 8-well chamber and then cultured in DMEM supplemented with 10% FBS, 100 U/mL penicillin, and 100 µg/mL of streptomycin at 37 °C, 5% CO_2_ until the chamber bottom reached 70% confluency. CD276Ab (200 μL) was added to the cells at 0, 12.5, 25.0, and 50.0 μg/mL, followed by subsequent incubation for 1 h at 37 °C, 5% CO_2_. After washing the cells three times with D-PBS(-), CD276Ab-PIC/m solution (200 μL, 10 μg/mL), the cells were incubated in the culture medium for 3 h at 37 °C. Subsequently, the cultured cell nuclei were counterstained with Hoechst 33258, then the cells were observed under a confocal laser scanning microscope (LSM780-06, Carl Zeiss, Oberkochen, Germany). For quantification, the fluorescence intensity of Cy5 within the cell cytoplasm (determined based on GFP fluorescence) was calculated per cell and compared amongst the different culture conditions (*n* = 50 per condition). All image analyses were conducted using Image J [29,30]. A significant difference in fluorescence intensity between culture conditions was detected by performing a Student’s *t*-test followed by Bonferroni correction for multiple comparisons.

## 3. Results and Discussion

### 3.1. Characterization of DBCO-CD276Ab

**Figure 2:**
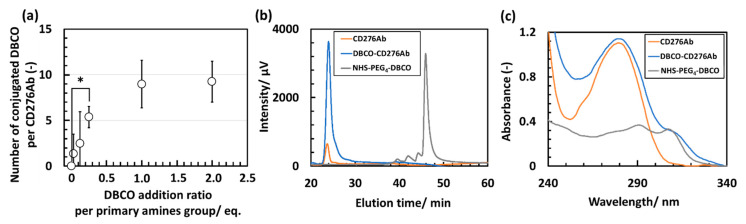
Characterization of DBCO-CD276Ab through fluorescamine assay, SEC, and UV absorption. (**a**) Number of DBCO conjugated to CD276Ab at various NHS-PEG_4_-DBCO addition concentrations, calculated using fluorescamine assay. N = 5. Statistical analysis was conducted via *t*-test, and the significant difference detected was represented by * *p* < 0.05. Data are shown in average ± standard error. (**b**) Chromatogram traces of CD276Ab (Orange), DBCO-CD276Ab (Blue), and NHS-PEG_4_-DBCO (Gray) measured by SEC (Detector: UV at 312 nm, flow rate: 0.5 mL/min, eluent: 10 mM PB (pH 7.4), 500 mM NaCl). (**c**) UV absorbance spectrum of CD276Ab (orange), DBCO-CD276Ab (blue), and NHS-PEG_4_-DBCO (gray).

The fabrication scheme of CD276Ab is described in the Supplementary Materials. To introduce CD276Ab to the micelle surface, PIC/m equipped with azide (N_3_) moiety (N_3_-PIC/m) and CD276Ab with dibenzocyclooctyne (DBCO) moiety conjugated to its primary amine groups (DBCO-CD276Ab) were prepared. Azide is known to react with DBCO at around pH 7.0 in an aqueous solution through click reaction [32,36]. The fabrication scheme of N_3_-PIC/m is described in the Supplementary Materials. To fabricate DBCO-CD276Ab at various surface DBCO densities, Dibenzocyclooctyne-poly (ethylene glycol)_4_-*N*-hydroxysuccinimidyl ester (NHS-PEG_4_-DBCO) was conjugated to the residual primary amine groups. DBCO-PEG_4_-NHS was added to the antibody solution at amounts equivalent to 0, 0.03, 0.125, 0.25, 1.0, and 2.0 times the amount of CD276Ab primary amine groups. The number of NHS-PEG_4_-DBCO conjugated to CD276Ab was then calculated by evaluating the number of primary amines remaining on the antibody and subtracting this from the initial amine count. Here, the number of amine groups per antibody was determined through a previously reported assay method using fluorescamine [26]. The number of NHS-PEG_4_-DBCO conjugated to CD276Ab increased with the increase in dose, reaching a maximum of 9 DBCO groups per antibody at 2.0 *eq*. feeding amount (Figure 2a). To accomplish the installation of CD276Ab to N_3_-PIC/m, one DBCO per CD276Ab is theoretically sufficient. To minimize the risk of CD276Ab denaturation, which can potentially cause negative impacts (e.g., a decrease in its activity, in vivo stability, and structural integrity) [37], CD276Ab conjugated with approximately five DBCOs (DBCO-PEG_4_-NHS feeding concentration of 0.25 *eq.* to primary amines) was chosen to be used for modifying N_3_-PIC/m. After removing the non-reacted NHS-PEG_4_-DBCO by Protein G column, the product was measured through size exclusion chromatography (SEC) (Figure 2b). The absorption of DBCO-CD276Ab was measured to check the conjugation of DBCO at 312 nm. The absorption spectrum of DBCO-CD276Ab consisted of a unique DBCO-derived peak, indicating successful conjugation of DBCO to CD276Ab (Figure 2c).

### 3.2. In Vitro Functionality Evaluation of DBCO-CD276Ab

**Figure 3:**
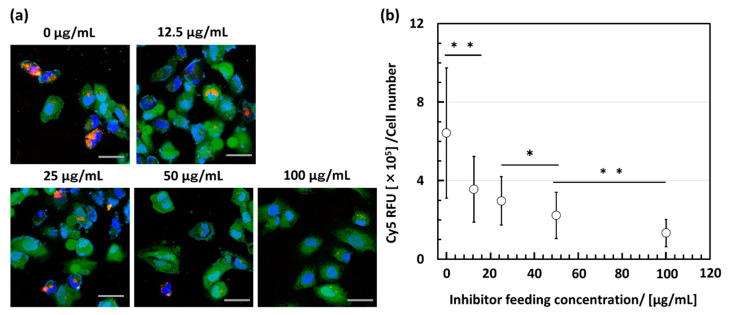
Inhibition assay conducted to validate the targeting ability of DBCO-CD276Ab. DAOY cells were incubated with both CD276Ab and DBCO-CD276Ab-Cy5. (**a**) CLSM images of DAOY cells incubated with DBCO-CD276Ab-Cy5 under inhibitor concentrations of 0, 12.5, 25, 50, and 100 μg/mL. Fluorescence derived from Hoechst 33258 counterstain (blue), GFP (green), and DBCO-CD276Ab-Cy5 (red) were observed to determine whether the antibodies could successfully target the CD276-expressing DAOY cells. The scale bar indicates 50 μm. (**b**) Quantitative analysis of Cy5-derived fluorescence observed within cells, conducted by measuring the brightness of each cell using ImageJ. For statistical analysis, a Student’s *t*-test followed by Bonferroni correction for multiple comparison was conducted. A significant difference is represented by * *p* < 0.05 and ** *p* < 0.01. Data are shown in average ± standard deviation.

To evaluate the functionality of the fabricated DBCO-CD276Ab, an inhibition assay using ‘*as prepared*’ CD276Ab and DBCO-CD276Ab was conducted. DAOY cells, derived from a pediatric brain tumor and known to express CD276 protein [13], were provided from ATCC (Manassas, VI, USA) and were gene-edited to express GFP to visualize the cell cytoplasm. DAOY cells were first incubated with abundant CD276Ab, incubated with Cy5-labeled DBCO-CD276Ab (DBCO-CD276Ab-Cy5, 2.2 Cy5 to 1 antibody), then imaged using a confocal laser scanning microscope (CLSM). The method of gene editing is described in detail in the Supplementary Materials. DAOY cells incubated without inhibition were also observed as a control. Under inhibition, DAOY cells indicated a low Cy5-derived signal within the cells. In contrast, those incubated without inhibition exhibited a noticeable accumulation of fluorescence within the cells (Figure 3a). This decrease in Cy5-derived fluorescence intensity under the existence of abundant inhibiting antibodies clearly indicates that DBCO-CD276Ab can selectively bind to its target, CD276. To further assess the effect of inhibition, inhibition was conducted under four conditions where the feeding concentration differed. The DBCO-CD276Ab-Cy5-derived fluorescence showed a significant decrease with an increase in inhibitor concentration (Figure 3b), which insists that the fabricated antibody competes with the inhibiting antibody when binding to CD276. These results strongly suggest that the targeting ability of the fabricated DBCO-CD276Ab was conserved throughout the fabrication process, with minimal denaturation or structural decay.

### 3.3. Preparation and Characterization of CD276Ab Conjugated Micelles

Model micelles, N_3_-PIC/m with cross-linked core polymers, were prepared based on a previous report [31]. Note that the comprising polymers were chemically cross-linked for stabilization so that any complication due to instability and disassembly can be excluded while characterizing the micelles. Briefly, the catiomer of Methoxy-PEG-poly[(5-aminopenthy)-*α*,*β*-aspartamide] (2k-72) and aniomer of Methoxy-PEG-poly(*α*,*β*-aspartic acid) (PEG-PAsp (*M*_n_ of PEG = 2000, DP of P(Asp) = 75)), PEG-PAsp-Cy5 (2.2k-73) and N_3_-PEG-PAsp (2k-68) were mixed in a phosphate buffer (10 mM, pH 7.4, 0 mM NaCl) at 1.0 mg/mL. The polymer solutions were stabilized by cross-linking the polymers with 1-ethyl-3-(3-dimethylaminopropyl) carbodiimide hydrochloride (EDC/HCl), then purified through ultracentrifugation.

DBCO-CD276Ab was used to modify the N_3_-PIC/m surface to enhance its delivery efficiency to CD276-expressing tumor cells. CD276 is a transmembrane protein highly expressed in tumor cells but absent or weakly expressed in normal tissue [38,39]. Thus, conjugation of CD276Ab to PIC/m surface was conducted to target tumor cells with minimal off-target effects. In addition, because the number of antibodies conjugated to the nanocarrier surface is known to influence the delivery efficiency [35], the number of CD276Ab that can be conjugated onto the PIC/m surface was investigated through fluorescence correlation spectroscopy (FCS). Upon FCS measurement, DBCO-CD276Ab labeled with DyLight 488 (1.2 DyLight 488 to 1 antibody) was introduced to the N_3_-PIC/m surface at various addition concentrations. The spherical morphology of the fabricated N_3_-PIC/m and CD276Ab conjugated PIC/m, which indicates successful fabrication and conjugation without causing disassembly, was confirmed via TEM imaging (Figure S4, see Supplementary Materials).

**Figure 4:**
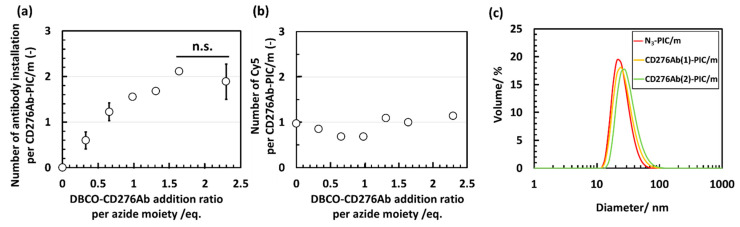
Characterization of CD276Ab-PIC/m after introducing CD276Ab by conjugating DBCO-CD276Ab to N_3_-PIC/m through click reaction at different addition ratios per azide moiety. (**a**) Number of antibodies installed to N_3_-PIC/m calculated from the counts per molecule obtained via FCS measurement (N = 3). Statistical analysis was conducted through Student’s *t*-test and n.s. indicates *p* > 0.05. Data are shown in average ± standard deviation. (**b**) Number of Cy5s per CD276Ab-PIC/m evaluated to check for aggregations. Representative size distribution was measured from (**c**) DLS measurement.

As the initial step of characterizing the CD276Ab installed PIC/m, the number of antibodies installed was measured by counts per molecule (evaluating the fluorescence derived from DyLight 488 conjugated on DBCO-CD276Ab). Results confirmed that 1.9 antibodies could be introduced by increasing the addition concentration to 2.3 *eq.* (Figure 4a). Furthermore, the number of Cy5 particles retained on CD276Ab-PIC/m was similar to that of naked N_3_-PIC/m regardless of the introduction of CD276Ab (Figure 4b). Therefore, the number of CD276Ab per PIC/m did not increase due to aggregation of the carriers; instead, the increase in number solely reflects the increase in the number of CD276Ab on the PIC/m surface. This is because steric hindrance affected to lower the CD276Ab conjugation efficiency [35,40,41]. Nevertheless, FCS measurements of the CD276Ab-PIC/m indicated successful installation of the antibody without causing disassembly of the micelle structure. However, it must be noted that the PIC/m used in this study has remarkably high stability compared to any drug-loaded micelles, which assemble through hydrophobic and/or ionic interactions. Thus, the effect of CD276Ab conjugation on the morphology of less robust micelles must be carefully considered when designing CD276Ab micelles that carry therapeutic drugs.

In addition to the FCS measurements described above, the particle size and polydispersity of the prepared samples were measured by dynamic light scattering (DLS) to confirm the validity of particle size and structure (Figure 4c). The measured particle size of the PIC/m modified with 1.2 CD276Ab (CD276Ab(1)-PIC/m) and 1.9 CD276Ab (CD276Ab(2)-PIC/m) were between 30–40 nm (Table 1), indicating that the change in particle size occurred due to the conjugation of the antibody. The polydispersity of the micelles was significantly increased after the introduction of CD276Ab compared to that before the introduction, presumably due to the structure changing. The polydispersity index can be represented using a derived moment $k_{2}$ [42] and decay constant Γ as:

(6) $$\text{Polydispersity index}=\frac{k_{2}}{\Gamma^{2}}$$

Here, decay constant Γ can be expressed as:

(7) $$\Gamma =(D_{T}q^{2}+{6D}_{R})$$

where $D_{T}$ is the translational diffusion coefficient and $D_{R}$ is the rotational diffusion coefficient. It is reported that $D_{R}$ decreases as the structure changes from a sphere to a prolate ellipsoid due to depolarized signals [43]. Such change in micelle morphology is suspected to be a cause of the increase in polydispersity index as the number of conjugated antibodies increased.

The zeta potential of CD276Ab and CD276Ab-PIC/m was measured, as it is a highly relevant parameter affecting the stability of nanocarriers during blood circulation. The obtained results demonstrated that CD276Ab has a weakly negative charge, consistent with previous reports [44]. In addition, N_3_-PIC/m, CD276Ab(1)-PIC/m and CD276Ab(2)-PIC/m displayed a negative zeta potential. Although a positive charge typically aids in the internalization of the nanocarriers, a negative charge could positively affect the carrier circulation due to the negatively charged luminal surface of blood vessels, which causes positively charged nanoparticles to be eliminated from circulation [45]. As a result, the zeta potential of the nanocarriers may exhibit the characteristics required for extended blood circulation.

### 3.4. Evaluation of Cell Internalization Efficiency of CD276Ab Decorated PIC/m

**Figure 5:**
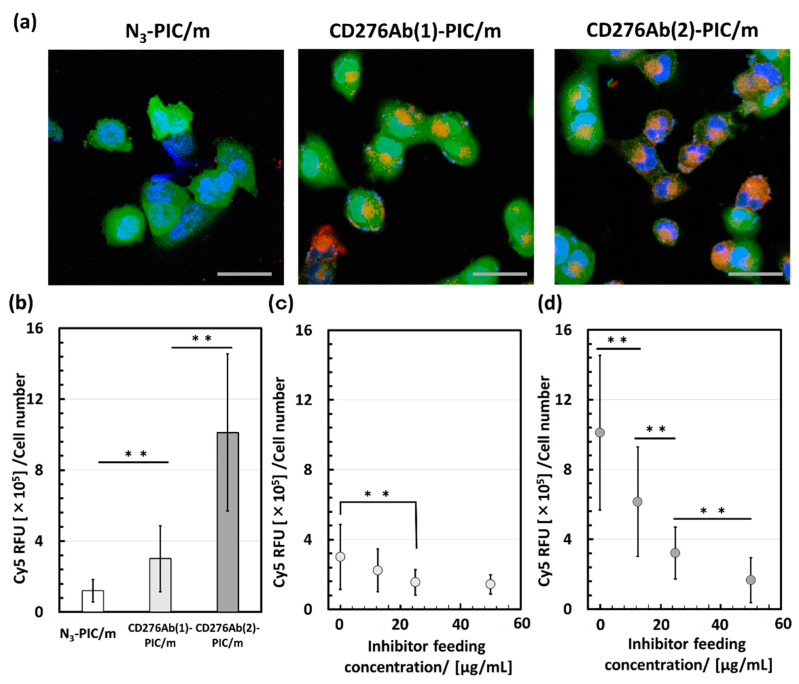
In vitro evaluation of internalization efficiency of CD276Ab installed micelles. (**a**) N_3_-PIC/m, CD276Ab(1)-PIC/m, and CD276Ab(2)-PIC/m were incubated with DAOY cells at 10 μg/mL (polymer concentration) for 3 h, then counterstained with Hoechst 33258. The fluorescence of Cy5 (red), GFP (green), and the nucleus (blue) were detected using a CLSM. The scale bar indicates 50 μm. (**b**) Cy5 fluorescence intensity within each cell was quantified by ImageJ (*n* = 50) and then tested for significant difference via a Student’s *t*-test followed by Bonferroni correction for multiple analyses. A significant difference was detected and represented as ** *p* < 0.001. Error bars indicate standard deviation. An inhibition assay was conducted for (**c**) CD276Ab(1)-PIC/m and (**d**) CD276Ab(2)-PIC/m. The Cy5 fluorescence intensity within each cell was quantified by ImageJ (*n* = 50) and then tested for significant differences via a Student’s *t*-test followed by Bonferroni correction for multiple comparisons. Significant difference was represented as ** *p* < 0.001. Error bars indicate standard deviation.

CD276Ab was conjugated onto the PIC/m surface to enhance the delivery efficiency to the CD276-expressing tumor cells. The cytotoxicity of the fabricated CD276Ab-PIC/m was first evaluated, where no significant increase in cytotoxicity due to the conjugation of CD276Ab was detected (Figure S5, see Supplementary Materials). To validate the effect of CD276Ab conjugation, CD276Ab conjugated PIC/m were incubated with DAOY cells known to express CD276, and their uptake was evaluated via the Cy5-derived fluorescence under a CLSM. While the Cy5-derived fluorescence was not visible in DAOY cells incubated with naked micelles (N_3_-PIC/m), a significant increase in internalization ratio was detected with both CD276Ab(1)-PIC/m and CD276Ab(2)-PIC/m (Figure 5a). This result suggests that installing a PIC/m surface with CD276Ab is an effective method in amplifying the delivery efficiency of micelles to DAOY cells and that the efficiency increases as the number of CD276Ab conjugated to the micelle surface increases (Figure 5b). This increase in cell uptake is presumably due to the multiple conjugated antibodies eliciting a multivalent binding effect [41,46,47]. In fact, a study showed that the degree of conjugation of anti-transferrin receptor (TfR) monoclonal antibody to Cylindrical poly(ethylene glycol)-based PRINT nanoparticles greatly affects the internalization rate of nanoparticles due to the multivalent binding that occurs between TfR and anti-TfR antibodies [48]. An inhibition assay was performed to check the potency and specificity of the CD276Ab installed PIC/m. Results exhibited a significant decrease in Cy5-derived fluorescence within DAOY cells with an increase in the concentration of the inhibiting CD276Ab (Figure 5c,d). Thus, it is safe to conclude that the CD276Ab-PIC/m is selectively recognizing CD276 and using this to enter the tumor cells. Together these results suggest that modifying the micelle surface with CD276Ab is effective in homing micelles specifically to CD276-expressing cancer cells at high efficiency by utilizing the immunological recognition function of CD276Ab. Although the results obtained thus far suggest that the conjugation of CD276Ab to micelle surface holds great promise for accumulation in tumors, it is imperative for such CD276Ab-installed micelles to first penetrate the blood-brain barrier (BBB) before they can access the tumor cells. While the WNT subtype medulloblastoma has been shown to have a leaky vasculature that may facilitate the penetration of PIC/m through the BBB, other subtypes (e.g., group 3, group 4, and Sonic hedgehog subtypes) have tumor cells barricaded by tight junctions between endothelial cells [49]. Therefore, future research efforts should prioritize strategies that enhance the BBB penetrability of these micelles through additional modifications to their surface, such as the installation of transferrin receptor antibodies.

## 4. Conclusions

In this study, an anti-CD276 antibody was introduced to the N_3_-PIC/m surface to enhance the delivery efficiency to pediatric brain tumor cells. Successful conjugation of DBCO-modified CD276Ab to N_3_-PIC/m was confirmed through FCS measurement, where one or two antibodies were introduced. The immunological recognition function of CD276Ab-PIC/m was demonstrated via an *in vitro* evaluation using CD276-expressing DAOY cells, where the cell internalization efficiency was significantly enhanced. The number of CD276Ab introduced was also shown to be an important factor, as CD276Ab(2)-PIC/m showed significantly higher efficiency compared to CD276Ab(1)-PIC/m, possibly due to the multivalent bonding that occurred between CD276 and CD276Ab. Our results indicate that installing CD276Ab on the micelle surface as a ligand to target tumor cells is an effective method for improving drug delivery efficiency to pediatric brain tumors.

## Figures and Tables

**Table 1:** Structural analysis by DLS and FCS before and after the introduction of CD276Ab to PIC/m surface.

	CD276Ab	N_3_-PIC/m	CD276Ab(1)-PIC/m	CD276Ab(2)-PIC/m
Diameter ^a, b^/nm (DLS)	-	30 ± 0.1	36 ± 0.9	45 ± 1.8
Polydispersity index ^a^	-	0.09 ± 0.02	0.20 ± 0.03	0.30 ± 0.03
Zeta potential ^c^/mV	−1.04 ± 0.40	−7.44 ± 1.53	−8.01 ± 0.53	−8.50 ± 2.09
Diameter ^a, d^/nm (FCS)	-	23 ± 0.6	34 ± 2.2	37 ± 0.8

^a^ Data shown in average ± standard deviation (N = 3). ^b^ Calculated using the cumulant method. ^c^ Data shown in average ± standard deviation (*n* = 3). ^d^ Calculated using the Einstein Stokes formula [34].

## Data Availability

Not applicable.

## Supplementary Materials

The following supporting information can be downloaded at: https://www.mdpi.com/article/10.3390/polym15071808/s1, Figure S1: Calibration curve showing the linear range of CD276Ab’s absorbance at 280 nm in relation to its concentration; Figure S2: Counter plot of DAOY cells with isotype and a PE-conjugated anti-CD276 antibody; Figure S3: Chromatogram traces of CD276Ab-PIC/m, CD276Ab-PIC/m, and DBCO-CD276Ab; Figure S4: TEM images of (**a**) N_3_-PIC/m, (**b**) CD276Ab(1)-PIC/m, and (**c**) CD276Ab(2)-PIC/m; Figure S5: Viability of DAOY cells incubated with N3-PIC/m, CD276Ab(1)-PIC/m, and CD276Ab(2)-PIC/m.

## References

[1] Ostrom Q.T., Gittleman H., Truitt G., Boscia A., Kruchko C., Barnholtz-Sloan J.S. (2018). CBTRUS Statistical Report: Primary Brain and Other Central Nervous System Tumors Diagnosed in the United States in 2011–2015. Neuro-Oncol..

[2] Shaik S., Maegawa S., Gopalakrishnan V. (2021). Medulloblastoma: Novel insights into emerging therapeutic targets. Expert Opin. Ther. Targets.

[3] Rudà R., Reifenberger G., Frappaz D., Pfister M.S., Laprie A., Santarius T., Roth P., Tonn C.J., Soffietti R., Weller M. (2018). EANO guidelines for the diagnosis and treatment of ependymal tumors. Neuro-Oncol..

[4] Pollack F.I., Agnihotri S., Broniscer A. (2019). Childhood brain tumors: Current management, biological insights, and future directions. J. Neurosurg. Pediatr..

[5] Tait M.D., Thornton-Jones H., Bloom J.H., Lemerle J., Morris-Jones P. (1990). Adjuvant chemotherapy for medulloblastoma: The first multi-centre control trial of the International Society of Paediatric Oncology (SIOP I). Eur. J. Cancer.

[6] Rutkowski S., Bode U., Deinlein F., Ottensmeier H. (2005). Treatment of Early Childhood Medulloblastoma by Postoperative Chemotherapy Alone. N. Engl. J. Med..

[7] Okada K., Yamasaki K., Tanaka C., Fujisaki H., Osugi Y., Hara H. (2013). Phase I Study of Bevacizumab Plus Irinotecan in Pediatric Patients with Recurrent/Refractory Solid Tumors. Jpn. J. Clin. Oncol..

[8] Hudson M.M., Ness K.K., Gurney G.J., Chemaitilly W., Krull R.K., Green M.D., Armstrong T.G., Nottage F.K., Sklar A.C., Srivastava K.D. (2013). Clinical Ascertainment of Health Outcomes Among Adults Treated for Childhood Cancer. JAMA.

[9] Petel P.J., Spiller E.S., Barker D.E. (2021). Drug penetration in pediatric brain tumors: Challenges and opportunities. Pediatr. Blood Cancer.

[10] Zucker D., Andriyanov V.A., Steiner A., Raviv U., Barenholz Y. (2012). Characterization of PEGylated nanoliposomes co-remotely loaded with topotecan and vincristine: Relating structure and pharmacokinetics to therapeutic efficacy. J. Control. Release.

[11] Sabina Q., Kataoka K., Cabral H. (2022). Nanomedicine for brain cancer. Adv. Drug Deliv. Rev..

[12] Majzner G.R., Therucath L.J., Nellan A., Heitzeneder S., Cui Y., Mount W.C., Rietberg P.S., Linde H.M., Xu P., Rota C. (2019). CAR T Cells Targeting B7-H3, a Pan-Cancer Antigen, Demonstrate Potent Preclinical Activity Against Pediatric Solid Tumors and Brain Tumors. Clin. Cancer Res..

[13] Li S., Poolen G.C., van Vliet L.C., Schipper J.G., Broekhuizen R., Monnikhof M., Van Hecke W., Vermeulen J.F., Bovenschen N. (2022). Pediatric medulloblastoma express immune checkpoint B7-H3. Clin. Transl. Oncol..

[14] Bao R., Wang Y., Lai J., Zhu H., Zhao Y., Li S., Li N., Huang J., Yang Z., Wang F. (2019). Enhancing Anti-PD-1/PD-L1 Immune Checkpoint Inhibitory Cancer Therapy by CD276-Targeted Photodynamic Ablation of Tumor Cells and Tumor Vasculature. Mol. Pharm..

[15] Wilson E.K., Bachwal V.S., Abou-Elkacem L., Jensen K., Machtaler S., Tian L., Willmann K.J. (2017). Spectroscopic Photoacoustic Molecular Imaging of Breast Cancer using a B7-H3-targeted ICG Contrast Agent. Theranostics.

[16] Wang Q., Kumar V., Lin F., Sethi B., Coulter W.D., McGuire R.T., Mahoto I.R. (2020). ApoE mimetic peptide targeted nanoparticles carrying a BRD4 inhibitor for treating Medulloblastoma in mice. J. Control. Release.

[17] Ruan S., Qin L., Xiao W., Hu C., Zhou Y., Wnag R., Sun X., Yu W., He Q., Gao H. (2018). Acid-Responsive Transferrin Dissociation and GLUT Mediated Exocytosis for Increased Blood–Brain Barrier Transcytosis and Programmed Glioma Targeting Delivery. Adv. Funct. Mater..

[18] Ruan S., Yuan M., Zhang L., Hu G., Chen J., Cun X., Zhang Q., Yang Y., He Q., Gao H. (2015). Tumor microenvironment sensitive doxorubicin delivery and release to glioma using angiopep-2 decorated gold nanoparticles. Biomaterials.

[19] Miura Y., Takenaka T., Toh K., Wu S., Nishihara H., Kano M.R., Ino Y., Nomoto T., Matsumoto Y., Koyama H. (2013). Cyclic RGD-linked polymeric micelles for targeted delivery of platinum anticancer drugs to glioblastoma through the blood-brain tumor barrier. ACS Nano.

[20] McQuigg D.W., Kaplan J.I., Dubin P.L. (1992). Critical conditions for the binding of polyelectrolytes to small oppositely charged micelles. J. Phys. Chem..

[21] Wang Y., Kimura K., Huang Q., Dubin P.L., Jaeger W. (1999). Effects of Salt on Polyelectrolyte-Micelle Coacervation. Macromolecules.

[22] Chen J.-X., Wang M., Tian H.-H., Chen J.-H. (2015). Hyaluronic acid and polyethylenimine self-assembled polyion complexes as pH-sensitive drug carrier for cancer therapy. Coll. Surf. B Biointerfaces.

[23] Bell B.J., Rink S.J., Eckerdt F., Clymer J., Goldman S., Shad Thaxton C., Platanias C.L. (2018). HDL nanoparticles targeting sonic hedgehog subtype medulloblastoma. Sci. Rep..

[24] Ishii M., Nakakido M., Caaveiro J., Kuroda D., Okumura C., Maruyama T., Entzminger K., Tsumoto K. (2021). Structural basis for antigen recognition by methylated lysine–specific antibodies. J. Biol. Chem..

[25] Tao A., Huang G., Igarashi K., Hong T., Liao S., Stellacci F., Matsumoto Y., Yamasoba T., Kataoka K. (2019). Polymeric Micelles Loading Proteins through Concurrent Ion Complexation and pH-Cleavable Covalent Bonding for In Vivo Delivery. Macromol. Biosci..

[26] Xu P., Kelly M., Vann F.W., Qadri F., Ryan T.E., Kováč P. (2017). Conjugate Vaccines from Bacterial Antigens by Squaric Acid Chemistry: A Closer Look. ChemBioChem.

[27] Dasari R., La Clair J.J., Kornienko A. (2018). Irreversible Protein Labeling by Paal–Knorr Conjugation. ChemBioChem.

[28] Manukyan G., Kriegova E., Slavik L., Mikulkova Z., Ulehlova J., Martiosyan A., Papajik T. (2023). Antiphospholipid antibody-mediated NK cell cytotoxicity. J. Reprod. Immunol..

[29] Ahn J., Miura Y., Yamada N., Chida T., Liu Z., Kim A., Sato R., Tsumura R., Koga Y., Yasunaga M. (2015). Antibody fragment-conjugated polymeric micelles incorporating platinum drugs for targeted therapy of pancreatic cancer. Biomaterials.

[30] Schneider C.A., Rasband W.S., Eliceiri K.W. (2012). NIH Image to ImageJ: 25 years of image analysis. Nat. Methods.

[31] Carter G., Liu X., Tochary A.T., Dirisala A., Toh K., Anraku Y., Kataoka K. (2020). Targeting nanoparticles to the brain by exploiting the blood–brain barrier impermeability to selectively label the brain endothelium. Proc. Natl. Acad. Sci. USA.

[32] Gai M., Simon J., Lieberwirth I., Mailänder V., Morsbach S., Landfester K. (2020). A bio-orthogonal functionalization strategy for site-specific coupling of antibodies on vesicle surfaces after self-assembly. Polym. Chem..

[33] Yang W., Miyazaki T., Chen P., Hong T., Naio M., Miyahara Y., Matsumoto A., Kataoka K., Miyata K., Cabral H. (2021). Block catiomer with flexible cationic segment enhances complexation with siRNA and the delivery performance in vitro. Sci. Technol. Adv. Mater..

[34] Loman A., Dertinger T., Koverling F., Enderlein J. (2008). Comparison of optical saturation effects in conventional and dual-focus fluorescence correlation spectroscopy. Chem. Phys. Lett..

[35] Min S.H., Kim J.H., Ahn J., Naito M., Hayashi K., Toh K., Kim S.B., Matsumura Y., Kwon C.I., Miyata K. (2018). Tuned Density of Anti-Tissue Factor Antibody Fragment onto siRNA-Loaded Polyion Complex Micelles for Optimizing Targetability into Pancreatic Cancer Cells. Biomacromolecules.

[36] Beck S., Schulze J., Räder H., Holm R., Schinnerer M., Barz M., Koynov K., Zental R. (2018). Site-Specific DBCO Modification of DEC205 Antibody for Polymer Conjugation. Polymers.

[37] Shen B., Xu K., Liu L., Raab H., Bhakta S., Kenrick M., Parsons-Reponte K.L., Tien J., Yu S., Mai E. (2012). Conjugation site modulates the in vivo stability and therapeutic activity of antibody-drug conjugates. Nat. Biotechnol..

[38] Zhang H., Zhang J., Li C., Xu H., Dong R., Chen C.C., Hua W. (2020). Survival Association and Cell Cycle Effects of B7H3 in Neuroblastoma. J. Korean Neurosurg. Soc..

[39] Huang B., Kuom L., Wang J., He B., Fenf R., Xian N., Zhang Q., Chen L., Huan G. (2020). B7-H3 specific T cells with chimeric antigen receptor and decoy PD-1 receptors eradicate established solid human tumors in mouse models. OncoImmunology.

[40] Florinas S., Liu M., Fleming R., Vlerken-Ysla V.L., Ayriss J., Glibreth R., Dimasi N., Gao C., Wu H., Xu Z. (2016). A Nanoparticle Platform to Evaluate Bioconjugation and Receptor-Mediated Cell Uptake Using Cross-Linked Polyion Complex Micelles Bearing Antibody Fragments. Biomacromolecules.

[41] Chen S., Florinas S., Teitgen A., Xu Z., Gao C., Wu H., Kataoka K., Cabral H., Christie J.R. (2017). Controlled Fab installation onto polymeric micelle nanoparticles for tuned bioactivity. Sci. Technol. Adv. Mater..

[42] Stetefeld J., Mckenna A.S., Pated R.T. (2016). Dynamic light scattering: A practical guide and applications in biomedical sciences. Biophys. Rev..

[43] Hoffmann M., Wagner S.C., Harnau L., Wittemann A. (2009). 3D Brownian Diffusion of Submicron-Sized Particle Clusters. ACS Nano.

[44] Filoti D.I., Shire S.J., Yadav S., Laue T.M. (2015). Comparative study of analytical techniques for determining protein charge. J. Pharm. Sci..

[45] Nel A.E., Mädler L., Velegol D., Xia T., Hoek E.M.V., Somasundaran P., Klaessig F., Castranova V., Thompson M. (2009). Unnderstanding biophysicochemical interactions at the nano-bio interface. Nat. Mater..

[46] Gilbreth N.R., Novarra S., Wetzel L., Florinas S., Cabral H., Kataoka K., Rios-Doria J., Christie R., Baca M. (2016). Lipid- and polyion complex-based micelles as agonist platforms for TNFR superfamily receptors. J. Control. Release.

[47] Miyazaki T., Chen S., Florinas S., Igarashi K., Matsumoto Y., Yamasoba T., Xu Z., Wu H., Gao C., Kataoka K. (2022). A Hoechst Reporter Enables Visualization of Drug Engagement In Vitro and In Vivo: Toward Safe and Effective Nanodrug Delivery. ACS Nano.

[48] Wang J., Tian S., Petros A.R., Napier E.M., DeSimone M.J. (2010). The Complex Role of Multivalency in Nanoparticles Targeting the Transferrin Receptor for Cancer Therapies. J. Am. Chem. Soc..

[49] Phoenix T.N., Patmore D.M., Boop S., Boulos N., Jacus M.O., Patel Y.T., Roussel M.F., Finkelstein D., Goumnerova L., Perreault S. (2016). Medulloblastoma genotype dictates blood brain barrier phenotype. Cancer Cell.

