# Supplementary material for: Ligand Installation to Polymeric Micelles for Pediatric Brain Tumor Targeting
Source: Polymers (Basel). 2023 Apr 6;15(7):1808. doi: 10.3390/polym15071808 (PMC10097392; doi:10.3390/polym15071808)
Supplement: Supplementary file 1 [file polymers-15-01808-s001.zip › polymers-2304596-supplementary.pdf]

Supplementary Materials: Ligand Installation to Polymeric Micelles for Pediatric Brain Tumor Targeting

Takayoshi Watanabe [1,†], Hayato Laurence Mizuno [1,2,†], Jumpei Norimatsu [1], Takumi Obara [1], Horacio Cabral [1], Kouhei Tsumoto [1,3,4], Makoto Nakakido [1,3], Daisuke Kawauchi [2,*], and Yasutaka Anraku [1,5,*]

[1] Department of Bioengineering, Graduate School of Engineering, The University of Tokyo, 7-3-1 Hongo, Bunkyo-ku, Tokyo 113-8656, Japan
[2] Department of Biochemistry and Cellular Biology, National Center of Neurology and Psychiatry (NCNP), Tokyo 187-8551, Japan
[3] Department of Chemistry and Biotechnology, School of Engineering, The University of Tokyo, Tokyo 113-8654, Japan
[4] Medical Proteomics Laboratory, The Institute of Medical Science, The University of Tokyo, Tokyo 113-8654, Japan
[5] Department of Materials Science and Engineering, School of Materials and Chemical Technology, Tokyo Institute of Technology, Tokyo 152-8550, Japan
* Correspondence: d.kawauchi@ncnp.go.jp (D.K.); anraku0915@g.ecc.u-tokyo.ac.jp (Y.A.)
† These authors contributed equally to this work.

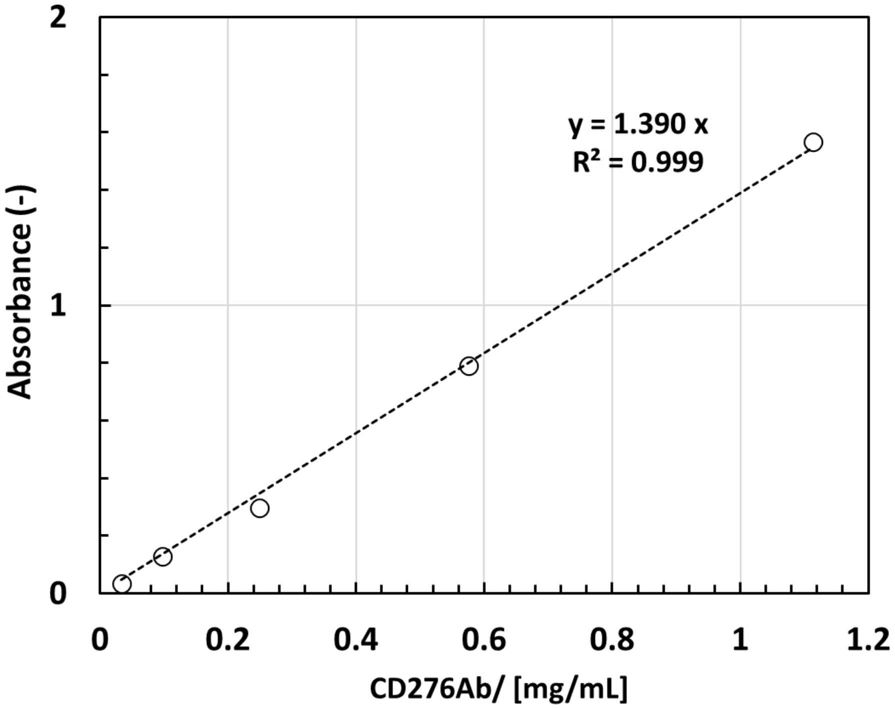

Figure S1. Calibration curve showing the linear range of CD276Ab's absorbance at 280 nm in relation to its concentration. Linearity was detected up to 1.1 mg/m with and R^2 of 0.999.

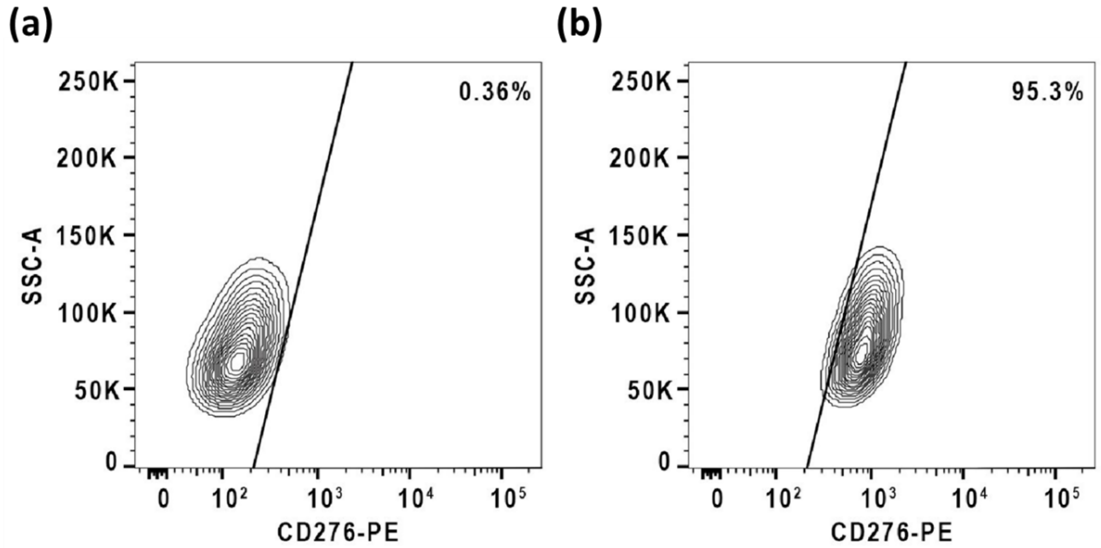

Figure S2. Counter plot of DAOY cells with isotype (A) and a PE-conjugated anti-CD276 antibody (B).

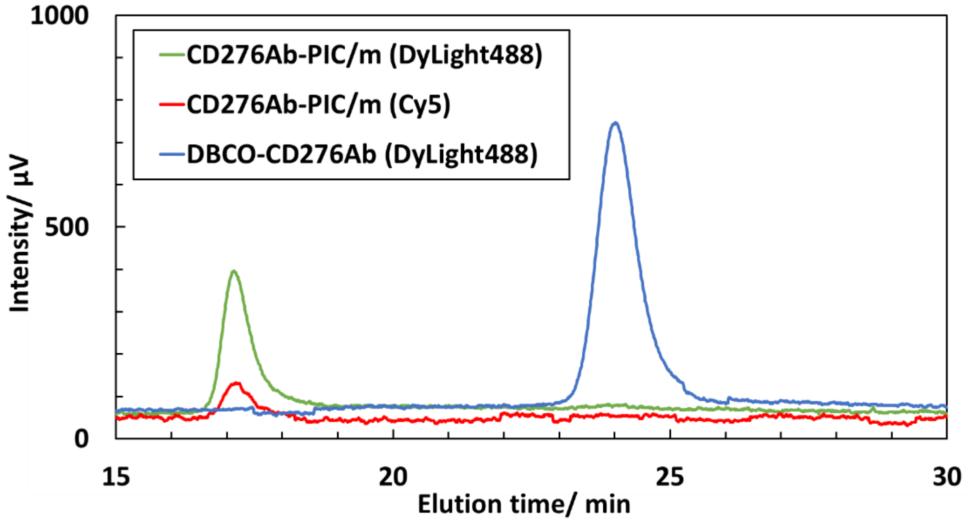

Figure S3. Chromatogram traces of CD276Ab-PIC/m (green), CD276Ab-PIC/m (red), and DBCO-CD276Ab (blue). The fluorescence of DyLight 488 conjugated to the CD275Ab was used for the detection of CD276Ab-PIC/m and DBCO-CD276Ab (Excitation: 493 nm, emission: 518 nm), while the fluorescence derived from Cy5 was used for the detection of CD276Ab-PIC/m (Excitation: 643 nm, emission: 667 nm). Flow rate: 0.5 mL/min, eluent: 10 mM phosphate buffer (pH 7.4), 500 mM NaCl.

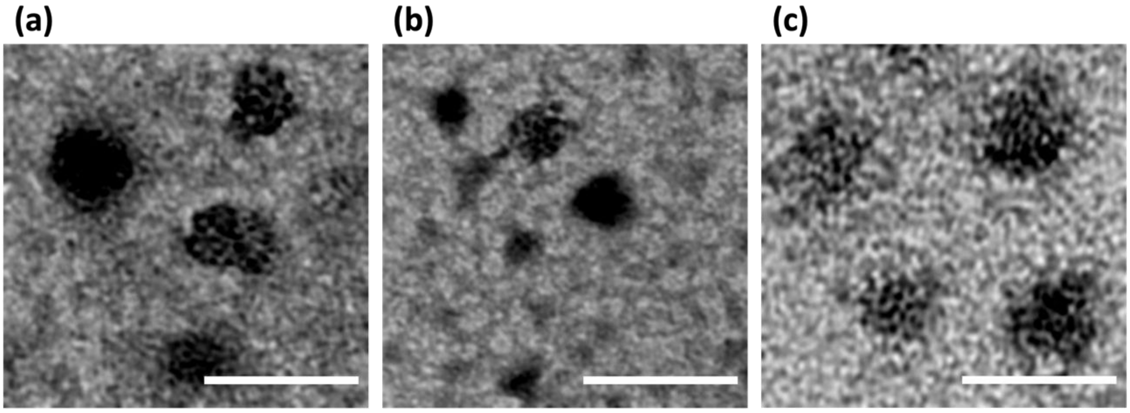

Figure S4. TEM images of (a) N_3-PIC/m, (b) CD276Ab(1)-PIC/m, and (c) CD276Ab(2)-PIC/m. Scale bars indicate 50 nm.

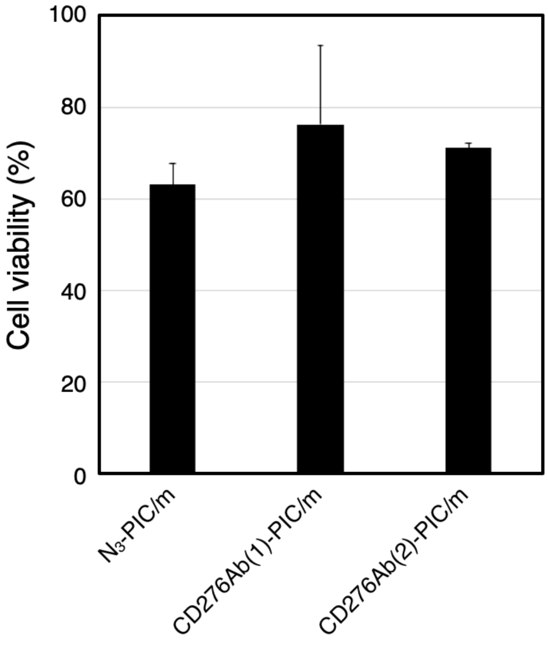

Figure S5. Viability of DAOY cells incubated with N_3-PIC/m, CD276Ab(1)-PIC/m, and CD276Ab(2)-PIC/m.
